# Antibiotic dispensing without a prescription across community pharmacies: A simulated patient study

**DOI:** 10.1016/j.rcsop.2025.100590

**Published:** 2025-03-13

**Authors:** Thu Anh Do, Phuong Bao Quan, Thy Tran-Bao Le, Tuyen Thanh Du, Suong Thi-Thanh Duong, Kim Thai-Thien Nguyen, Khoi Ngoc Nguyen, Hoa Quoc Nguyen

**Affiliations:** a Faculty of Pharmacy, University of Medicine and Pharmacy at Ho Chi Minh City, Ho Chi Minh City, Viet Nam; b School of Pharmacy, Queen's University Belfast, Belfast, United Kingdom

**Keywords:** Antibiotics, Community pharmacists, Pharmacies, Common cold, Simulated patient

## Abstract

**Objectives:**

This study aimed to investigate the proportion of antibiotics dispensed, counseling content, and factors associated with antibiotic dispensing for patients with common cold symptoms among community pharmacists (CPs) in Ho Chi Minh City (HCMC).

**Methods:**

A cross-sectional study was conducted from January to June 2023 using a simulated patient (SP) method. A total of 352 community pharmacies across 24 districts in HCMC were randomly selected. The SP provided complaints including having fever, sore throat, cough, swallowing difficulty, stuffy nose and asked for treatment. Bayesian Model Averaging was undertaken to determine the factors associated with antibiotic dispensing practices.

**Results:**

Of 352 enrolled pharmacies, the proportions of antibiotics suggested and eventually dispensed by CPs were 86.4 % and 83.0 %, respectively. Antipyretics, corticosteroids, and cough medicines were also frequently dispensed (94.9 %, 90.1 %, and 85.8 %, respectively). Only 1.7 % of the CPs provided reasons to take antibiotics, and 8.8 % advised to take the full course of antibiotics. While 80.1 % of CPs counseled on the dosage of medications dispensed, 1.7 % provided information regarding potential drug adverse reactions. CPs at chain pharmacies and in urban areas were less likely to dispense antibiotics without a prescription than their peers at independent pharmacies (OR = 0.32; 95 % CI: 0.17–0.59) and in rural areas (OR = 0.53, 95 % CI: 0.29–0.97).

**Conclusion:**

A high proportion of CPs dispensing antibiotics without a prescription along with inadequate counseling content, especially at independent pharmacies and in rural areas was recorded. These issues hightlight the need to improve CPs' knowledge regarding antibiotic resistance and appropriate antibiotic use.

## 1 Introduction

Antimicrobial resistance (AMR) has been a top health concern with an estimated 4.95 million deaths associated with bacterial AMR in 2019.^1^ Several underlying drivers may contribute to development of AMR, including inappropriate use of antimicrobials, lack of awareness of AMR, and lack of rigorous laws and enforcement, such as dispensing antibiotics without a prescription.^2^ Dispensing antibiotics without a prescription may lead to misuse of antibiotics that contribute to the emergence and spread of AMR.^3^ Previous studies have reported the proportions of antibiotic dispensing without a prescription were above 60 %.^4^, ^5^, ^6^ Moreover, a study conducted across low- and middle-income countries also found that 35 % of consumers in six countries purchased antibiotics without prescriptions.^7^

In Vietnam, the Ministry of Health issued a Decision of “Regulations on Prescription in Outpatient Treatment” since 2008 and clearly stated that antibiotics must be dispensed with prescriptions.^8^^,^^9^ However, a survey conducted in Ha Noi in 2010 revealed that most pharmacists in both urban (88 %) and rural (91 %) areas distributed antibiotics without a prescription.^10^ After the “National Action Plan on Combating Drug Resistance in the Period 2013–2020” was issued in June 2013 by the Ministry of Health, the government promulgated Decree 176/2013/ND-CP in November 2013, stating that community pharmacists (CPs) would be fined $8 to $21 per infraction by local health authorities if they dispensed antibiotics without a prescription.^11^ Nevertheless, a national survey of 350 private pharmacies reported that more than 80 % of participants had been able to purchase antibiotics without a prescription.^12^ Subsequently, Decree 117/2020/ND-CP was enacted to replace Decree 176/2013/ND-CP with the fine increased to $200 to $400 for this violation.^13^

Since the publication of the new Decree, limited studies have been conducted to explore the dispensing practices of antibiotics at community pharmacies in Vietnam. Therefore, this study was conducted to investigate the proportion of antibiotics dispensed without a prescription, along with counseling content among CPs in Ho Chi Minh City (HCMC), Vietnam. We also aimed to identify factors associated with antibiotic dispensing without a prescription.

## 2 Methods

This was a cross-sectional study conducted from 1st January to 30th May 2023, using the simulated patient (SP) method.

### 2.1 Sampling

We aimed to approach community pharmacies across all districts of HCMC (13 urban and 11 rural), which was the largest city in Vietnam with a population of 9.4 million people in 2022.^14^

The list of registered Good Pharmacy Practice (GPP) pharmacies in 2022 was provided by the Department of Health of HCMC.^15^ GPP is a set of required standards for a community pharmacy to ensure the quality of medication dispensing for relevant clients.^16^ These standards include requirements for personnel, facilities, medication purchasing, dispensing, and storage. Based on a previous study,^17^ the identified sample size of our study was 352 pharmacies. The number of pharmacies visited by the SP in each district was allocated based on the proportion of pharmacies per district. All potential pharmacies were randomly assigned a number using a computer and arranged in order. Pharmacies which met the exclusion criteria were replaced by the next ones in sequence. Independent or chain pharmacies within the list of retail pharmacies were included in the study.^15^ Exclusion criteria comprised pharmacies with inactive status, hospital pharmacies, herbal/traditional medicine pharmacies, and pharmacies with non-pharmacist staff.

### 2.2 Study design and data collection

The SPs consisted of a pharmacist and two undergraduate pharmacy students. Each SP presented symptoms commonly associated with a mild upper respiratory tract infection, such as the common cold based on the specific scenario, which was developed by the research team and described in a previous publication,^18^ and evaluated by three experts in pharmacy practice (Table 1)**.** The symptoms presented were consistent with a common cold—one of the most frequent and self-limiting conditions encountered in the community —and were intentionally designed to reflect cases that typically did not warrant antibiotic use. To ensure consistency in data collection and minimize the Hawthorne effect, the SPs participated in practice sessions until they could provide consistent descriptions of the symptoms and questions in every scenario before visiting enrolled pharmacies. One pharmacist at each enrolled pharmacy was approached by two researchers: one acted as an SP, presenting symptoms and requesting medications, while the other observed and took notes. The SP only provided further information if asked by the CP. All medications were dispensed based on the CP's recommendation, and the SP did not request any specific medications, including antibiotics. Based on the scenario, the SP asked the CP to dispense medications for a two-day trial, and afterward would return to receive the full course if advised by the CP. After the encounter was over, the SP engaged in another scenario where she portrayed a relative interested in becoming a pharmacist and asked the CP further information regarding their educational level and years of experience. The SP then filled out the collection form within 15 min after leaving the pharmacy.

**Table 1:** Scenario of the SP.

Enquiries by the pharmacists	SP's response
Patient information	Female, 20 years old, average BMI.
Symptoms	Slight fever, dry cough, nasal decongestion, sore throat, trouble swallowing.
Duration of symptoms	On the 2nd day of symptoms.
Medical history	No allergies to any foods or medicines. No other diseases; COVID-19 negative self-test result. No pregnancy or breastfeeding. No other treatment taken.

The collected data comprised characteristics of CPs and pharmacies, medications dispensed (including antibiotics), CPs' enquiries, and counseling content. A pharmacy location was categorized as either “alley” or “street” based on the address details. Addresses containing a slash (‘/’) were classified as alleys. In addition, a pharmacy was considered a chain pharmacy if it was run by a corporation and operated in compliance with the rules of the corporation. It was anticipated that CPs, particularly those with extensive experience, might not consistently provide precise details about their years of practice, instead offering estimated ranges such as ‘more than 10 years’ or ‘over 20 years’.As a result, a 10-year cut-off was selected based on the nature of datacollected. The list of registered pharmacies in 2022 provided by the Department of Health of HCMC was also used to check if the encountered CPs were pharmacists-in-charge.^15^

### 2.3 Statistical analysis

The collected data was analyzed using R version 4.2.0. To compare differences in characteristics of CPs and proportion of antibiotic dispensing between pharmacies located in urban and rural areas, the chi-square test or Fisher's exact test was performed. A difference was considered significant if the *p*-value was less than 0.05.

Factors associated with antibiotic dispensing practices by CPs were also explored. Several variables were selected according to previous studies,^10^^,^^18^, ^19^, ^20^ including the geographic area (urban or rural), location (street or alley), type of pharmacy (independent or chain), CPs' gender (male or female), educational level (SDPharm, CDPharm, or Bpharm), years of experience (< 10 years or ≥ 10 years), and whether the CP was the pharmacist-in-charge. To identify if these variables were independent, Pearson's chi-square test was used to determine Phi or Cramer's V coefficients. The two variables were considered independent if the correlation coefficient was less than 0.5.^21^ Subsequently, the Bayesian Model Averaging (BMA) approach was used to identify suitable regression models for independent variables associated with antibiotic dispensing practices.^22^^,^^23^ Models and variables which had posterior probabilities >10 % were selected for the development of a final logistic regression model.

### 2.4 Ethical considerations

The study was approved by the Research Ethics Committee of the University of Medicine and Pharmacy at HCMC on November 11th, 2022 (Approval No: 860/HDDD-DHYD).

## 3 Results

### 3.1 General characteristics

Of 498 pharmacies randomly selected from the list of retail pharmacies, 143 did not meet the inclusion criteria (127 were not found due to wrong address or information details, 8 were integrated with private medical clinics, 3 were permanently closed, 3 were located in residential areas requiring access cards, and 2 were adjacent to pharmacies previously investigated). Of 355 pharmacies visited by the SPs, 3 were excluded as the sellers were not pharmacists.

Among 352 enrolled pharmacies, 156 (44.3 %) were in urban districts. Regarding characteristics of 352 CPs, 41 (11.6 %) were pharmacists-in-charge, 151 (45.8 %) had been working for more than 10 years, and 262 (51.1 %) had a Bachelor of Pharmacy degree. There were no significant differences between urban and rural areas regarding type of pharmacy (*p* = 0.218), gender (*p* = 0.245), educational level (*p* = 0.276), experience (*p* = 0.374) of the pharmacists, and pharmacist-in-charge (*p* = 0.617). (Table 2).

**Table 2:** Characteristics of pharmacies and community pharmacists.

Characteristics		Urban (n, %)	Rural (n,%)	Total (n, %)	p – value⁎
Geographical area (*n* = 352)		156 (44.3)	196 (55.7)	352 (100.0)	
Location (*n* = 352)	Alley	10 (6.4)	4 (2.0)	14 (4.0)	0.053
	Street	146 (93.6)	192 (98.0)	338 (96.0)	
Type of pharmacy (n = 352)	Independent	112 (71.8)	149 (76.0)	261 (74.1)	0.218
	Chain	44 (28.2)	47 (24.0)	91 (25.9)	
Pharmacist-in-charge (n = 352)	Yes	20 (12.8)	21 (10.7)	41 (11.6)	0.617
	No	136 (87.2)	175 (89.3)	311 (88.4)	
Gender (n = 352)	Female	135 (86.5)	160 (81.6)	295 (83.8)	0.245
	Male	21 (13.5)	36 (18.4)	57 (16.2)	
Educational level (*n* = 317)	SDPharm^a^	17 (13.1)	25 (13.4)	42 (13.2)	0.276
	CDPharm^b^	40 (30.8)	73 (39.0)	113 (35.6)	
	BPharm^c^	73 (56.2)	89 (47.6)	162 (51.1)	
Experience (*n* = 330)	< 10 years	80 (57.1)	99 (52.1)	179 (54.2)	0.374
	≥ 10 years	60 (42.9)	91 (47.9)	151 (45.8)	

⁎ Chi-square test; p < 0.05: statistically significant.

a Secondary Diploma in Pharmacy (after completing a 2-year Pharmacy education program).

b College Diploma in Pharmacy (after completing a 3-year Pharmacy education program).

c Bachelor of Pharmacy (after completing a 5-year Pharmacy education program).

### 3.2 Medicine dispensing

The most commonly dispensed drug class was antipyretics (94.9 %), followed by corticosteroids (90.1 %), cough medicines (85.8 %) and antibiotics (83.0 %). The frequencies of each drug class are presented in **Appendix S1.**

Of 352 CPs, 304 (86.4 %) suggested antibiotics for the SPs. However, after the SP asked for a two-day trial course, 12 CPs refused to dispense antibiotics for a duration of less than 3 days due to their perspective on inadequate treatment duration. Therefore, 292 (83.0 %) CPs eventually dispensed antibiotics (Table 3). Among the 12 pharmacists who suggested but did not dispense antibiotics, two were working in a rural area and three were working in independent pharmacies.

**Table 3:** The proportion of pharmacists counseling and eventually dispensing antibiotics.

Antibiotics	Urban n_1_ = 156 (n,%)	Rural n_2_ = 196 (n,%)	Total N = 352 (n,%)	p – value⁎
Counseled	132 (84.6)	172 (87.8)	304 (86.4)	0.436
Dispensed	122 (78.2)	170 (86.7)	292 (83.0)	0.045

⁎ Chi-square test; p < 0.05: statistically significant.

Of antibiotics dispensed by 292 CPs, cephalosporins were the most popular (144/292, 49.3 %), followed by penicillins (31.8 %) and fluoroquinolones (12.7 %). Overall, a total of 23 antibiotic agents were dispensed, including amoxicillin/ clavulanic acid (67/292, 67 %), cefalexin (13.4 %), cefixime (11.3 %), and cefuroxime (11.0 %). (**Appendix S2**).

A total of 285 (97.6 %) CPs dispensed one antibiotic agent, while the remaining 7 CPs (2.4 %) dispensed two antibiotic agents. Among the antibiotic combinations, the most common were cephalosporins and fluoroquinolones. (**Appendix S3**).

Of those enquiries provided by the CPs (**Appendix S4**), the most common were comorbidities (131/352, 37.2 %), patient identity (23.6 %) and allergy history (13.9 %).

Of 292 CPs who dispensed antibiotics, only 31 (10.6 %) advised the SPs to purchase a full course of antibiotics (according to their perspective): a minimum duration of 3 days (20 CPs), and a duration of 5 to 7 days (11 CPs). Additionally, 6 (2.1 %) CPs explained the use of antibiotics by stating that “sore throat is a symptom of bacterial infections that requires antibiotic treatment”.

Of the counseling content (**Appendix S5**), the most common were medication dosage (282/352, 80.1 %), route of administration (42.3 %), non-pharmacological approaches (41.5 %), and duration of treatment (34.9 %). Moreover, only 6 CPs (1.7 %) provided reasons to take antibiotics, and 31 (8.8 %) advised to take the full course of antibiotics.

### 3.3 Factors associated with antibiotic dispensing practices

Fully available variables from 307/352 CPs were used to identify those potentially associated with antibiotic dispensing practices. All predictor variables were considered independent due to negligible or weak correlations (**Appendix S6**).

The geographic area and type of pharmacy, which were predictor variables identified through the BMA approach, were used in the logistic regression analysis (**Appendix S7, S8**). CPs at chain pharmacies and in urban areas were less likely to dispense antibiotics than their peers at independent pharmacies (OR = 0.32; 95 % CI: 0.17–0.59) and in rural areas (OR = 0.53; 95 % CI: 0.29–0.97).

## 4 Discussion

In our study, antibiotics were highly obtainable in both urban and rural areas in HCMC (78.2 % and 86.7 %, respectively). Inadequate counseling practices of CPs regarding medications for the common cold were also recorded. In addition, the geographical area and type of pharmacy were factors significantly associated with dispensing antibiotics without a prescription.

Overall, 83.0 % of CPs eventually dispensed antibiotics without a prescription. This high proportion was roughly similar to figures recorded by previous studies in Vietnam (approximately 90 %) before 2020 when the new Decree regarding administrative sanctions for pharmacy practice was enacted.^10^^,^^19^ Despite the 20-fold increase in the fine for medication dispensing without a prescription since 2013, this penalty did not seem to prevent this inappropriate practice.^11^^,^^13^ Furthermore, a common cold is a viral upper respiratory tract infection, for which antibiotic treatment is not recommended.^24^, ^25^, ^26^, ^27^, 28. However, 6 CPs in our study advised the SPs that this condition was a bacterial infection that should have been treated with antibiotics. Subsequently, this highlights the importance of training programs to improve CPs' awareness of common infectious diseases. A previous study in Ha Noi in Vietnam showed that 27 % and 24 % of drug sellers in urban and rural areas, respectively, admitted that lack of knowledge was the reason for their inappropriate dispensing of antibiotics.^10^

Cephalosporins, penicillins, and fluoroquinolones were the most commonly dispensed antibiotic classes. The inappropriate use of these broad-spectrum antibiotics not only disrupts the intestinal microbiome, increasing the risk of adverse drug reactions and *Clostridioides difficile* infection, but also accelerates the development of AMR.^29^, ^30^, ^31^ In Vietnam, overuse of penicillins and fluoroquinolones has led to relatively low bacterial susceptibility, compounding the AMR challenge.^30^^,^^32^, ^33^, ^34^ According to the Essential Medicines List Antibiotic Book by the World Health Organization, fluoroquinolones were classified as the Watch group, which were considered to be used only for patients with severe clinical symptoms.^35^ Inappropriate use of fluoroquinolones may not only increase the risk of AMR but also adverse events such as tendon swelling or injury.^36^ The dispensing of tinidazole and rifampicin was also recorded, despite their restricted use being limited to specific infections, such as protozoal infections or tuberculosis.^37^^,^^38^ Rifampicin is an essential medicine to treat tuberculosis, and it is presented in all current tuberculosis treatment regimens published by Vietnam Ministry of Health.^39^ Consequently, the misuse of antibiotics in HCMC needs to be supervised more strictly to reduce the risk of AMR and unintentional harm from the use of unnecessary antibiotics. We also recorded that in the transaction with the highest price, the CP dispensed 9 medications (including a combination of penicillin and cephalosporin along with dietary supplements and various cough medicines). Therefore, this shows that polypharmacy can lead to inapproriate usage and increased patient cost of medications.

Besides antibiotics, our study also recorded other medications dispensed without a prescription, such as oral corticosteroids (90.1 %), and prescription non-steroidal anti-inflammatory drugs (NSAIDs) (21.3 %). Using corticosteroids and certain NSAIDs such as diclofenac, meloxicam, piroxicam, and celecoxib are not encouraged to treat the common cold due to lack of evidence.^24^^,^^25^^,^^40^ Moreover, inappropriate use of NSAIDs may cause some adverse drug reactions including gastrointestinal bleeding, cardiovascular and renal effects,^41^ while short-term usage of corticosteroids may also increase the rates of sepsis, venous thromboembolism, and fracture.^42^ A previous study in Ha Noi, Vietnam, showed that the rate of pharmacists dispensing oral corticosteroids without a prescription was 76 %, although they were aware of their malpractice and potential adverse drug reactions of these medications.^43^ Therefore, these issues highlight the need to implement stricter regulations and enhance pharmacist education to ensure the rational and safe use of these medications.

Regarding counseling content of the 352 CPs, while 304 (86.4 % of the CPs) suggested antibiotics to the SPs, only 31 (8.8 %) advised to take a full course of antibiotics. Moreover, it was inappropriate when 20 out of 31 CPs advised the SPs that a full course of antibiotics was only 3 days. According to the WHO AwaRe antibiotic book, the three-day treatment duration of oral antibiotics is recommended only for adult patients in specific infectious conditions, such as using amoxicillin for oral and dental infections or sulfamethoxazole/trimethoprim for lower urinary tract infection.^44^ This suggests that while some CPs were concerned about completing a full course of antibiotics, they lacked knowledge about the appropriate use of these medications. The percentage of CPs who counseled about medication dosage in our study was higher than that in the previous study of Zawahir et al. (2022) in Vietnam (80.1 % and 69.1 %, respectively).^19^ In contrast, only 42.3 % of the CPs provided instructions on medication route of adminstration, compared to 75.3 % reported by Zawahir et al. (2022).^19^ Moreover, a significantly low proportion (1.7 %) of CPs informed patients about side effects of medications. These inadequate counseling practices of the CPs undermined the GPP standards issue by the Ministry of Health.^16^ A study in Can Tho City, Vietnam, reported that CPs at GPP-certified pharmacies also dispensed antibiotics without a prescription and their counseling skills required improvement.^45^ Therefore, additional training courses are necessary to enhance the counseling abilities of CPs.^45^, ^46^, ^47^

In our study, CPs working in chain pharmacies were less likely to dispense antibiotics than their peers in independent pharmacies (OR = 0.32; 95 % CI: 0.17–0.59), which was similar to a review reported by Li Jinxi et al.(2023).^4^ This relationship may be attributed to two factors: first, employees at chain pharmacies were not directly benefiting when selling prescription medicines; and second, they might lose their jobs due to non-adherence to the chain's rules.^48^ In our study, the odds of antibiotics dispensed without a prescription diminishes by 47 % if the pharmacy was located in an urban area (OR = 0.53; 95 % CI: 0.29–0.97). This was possibly due to the fact that CPs in urban areas may have better knowledge of antibiotics and AMR. It was previously reported that drug sellers in rural areas had less knowledge regarding antibiotics and antibiotic resistance compared to their urban peers.^10^ Similar to previous studies, gender, educational level, and years of experience of the CPs were not related to the proportion of antibiotic dispensing in our study.^18^^,^^49^ A study by Do Thi Thuy Nga et al. (2014) reported that 100 % and 69 % of Vietnamese CPs in urban and rural areas admitted that they dispensed antibiotics due to fear of losing customers.^10^

Our study highlights the inappropriate antibiotic dispensing practices of CPs. Despite government efforts to curb the sale of prescription medications without a prescription by increasing fines for this violation, additional measures are needed to improve CPs' awareness on appropriate antibiotic use and improve their counseling skills, such as educational interventions, strict supervision, and stronger law enforcement.^10^^,^^19^^,^^46^^,^^47^ Research shows that multifaceted interventions involving law enforcement, education, and peer influence effectively improve practices in community pharmacies.^20^ Additionally, authorities can monitor medication sales through pharmacy management software to implement targeted educational interventions.^19^

To the best of our knowledge, this was the first study on the dispensing practice of antibiotics at community pharmacies in all districts of Ho Chi Minh City, Vietnam conducted after the publication of the new Decree (Decree No.117/2020/ND-CP). By using the simulated patient method, it was expected to reduce the Hawthorne effect which could have occurred when the CPs were aware of being observed and changed their practices and behaviors.^50^ However, some useful information from the CPs, such as their participation in Continuing Medical Education courses and underlying reasons to suggest antibiotics, was unable to be collected. Additionally, we could not investigate all the pharmacies in HCMC which may provide further insights regarding antibiotic dispensing without a prescription, despite our effort of pharmacy sampling across all districts.

## 5 Conclusion

Up to 83.0 % of the CPs in HCMC dispensed antibiotics without a prescription, along with inadequate counseling practices. In addition, CPs at independent pharmacies and in rural districts were more likely to sell antibiotics without a prescription. The authorities may need to supervise practices of CPs more strictly and enhance further approaches to raise the CPs' awareness about antibiotic resistance and appropriate antibiotic use.

## Funding

This research was funded by the University of Medicine and Pharmacy at Ho Chi Minh City under contract number 180/2022/HĐ-ĐHYD, dated 15/09/2022.

## CRediT authorship contribution statement

**Thu Anh Do:** Writing – review & editing, Writing – original draft, Visualization, Software, Resources, Methodology, Investigation, Formal analysis, Data curation, Conceptualization. **Phuong Bao Quan:** Writing – original draft, Resources, Investigation, Data curation, Conceptualization. **Thy Tran-Bao Le:** Writing – original draft, Visualization, Software, Resources, Methodology, Investigation, Conceptualization. **Tuyen Thanh Du:** Writing – review & editing, Resources, Investigation, Conceptualization. **Suong Thi-Thanh Duong:** Writing – review & editing, Resources, Investigation, Conceptualization. **Kim Thai-Thien Nguyen:** Writing – review & editing, Project administration, Conceptualization. **Khoi Ngoc Nguyen:** Writing – review & editing, Validation, Supervision, Methodology, Conceptualization. **Hoa Quoc Nguyen:** Writing – review & editing, Validation, Supervision, Project administration, Methodology, Conceptualization.

## Declaration of competing interest

The authors declare that they have no known competing financial interests or personal relationships that could have appeared to influence the work reported in this paper.
